# Residential radon exposure and lung cancer histology and stage: a population-based ecological study in Central Germany

**DOI:** 10.1007/s00432-026-06524-7

**Published:** 2026-05-31

**Authors:** Philipp Ernst, Tobias Rachow, Sebastian Henn, Jakob Friedrich Hammersen, Silvio Dittrich, Annika Heßmer, Alexander Hillig, Astrid Heßmer, Andreas Hochhaus

**Affiliations:** 1 Klinik für Innere Medizin II, Universitätsklinikum Jena, Comprehensive Cancer Center Central Germany, Campus Jena, Am Klinikum 1, 07740 Jena, Germany; 2 IOGP MVZ GmbH, Gera, Germany; 3 Institut für Wirtschaftsgeographie, Friedrich-Schiller-Universität Jena, Jena, Germany; 4 Wismut-Erbe-Forschung, Sächsische Akademie für Wissenschaft, Leipzig, Germany; 5 Landeskrebsregister Thüringen, Jena, Germany

**Keywords:** Lung cancer, Epidemiology, Radon exposure

## Abstract

**Purpose:**

Residential radon exposure is recognised as one of the leading environmental risk factors for lung cancer and acts synergistically with tobacco smoking. Thuringia, Germany, exhibits high geogenic radon potential; however, population-level associations with lung cancer histology and stage at diagnosis remain insufficiently characterised.

**Methods:**

We conducted a retrospective ecological analysis of 893 lung cancer cases (2018–2022) from the Thuringian State Cancer Registry. Cases from 96 communities were classified as high- or low-radon exposure based on geogenic radon potential and soil radon activity. Demographics, histology and stage were compared between groups.

**Results:**

Population-level lung cancer incidence did not differ significantly between low- and high-radon communities, consistent with age- and sex-adjusted Poisson regression showing no association with residential radon exposure (adjusted IRR 1.05, *p* = 0.473). However, all cases in women younger than 50 years occurred in high-radon communities. Adenocarcinoma was nominally more frequent (56% vs. 48%, *p* = 0.04), and among patients with small cell lung cancer (SCLC), extensive-stage disease was more common (64% vs. 49%, *p* = 0.04), with combined SCLC occurring exclusively; however, these findings were not significant after correction for multiple testing.

**Conclusion:**

In this ecological analysis, residential radon exposure was not associated with annual lung cancer incidence but was linked to tumour histology and stage at diagnosis. Given the ecological design and lack of smoking and occupational exposure data, these findings are hypothesis-generating and warrant further investigation to clarify radon’s role in lung cancer biology and prevention.

**Supplementary Information:**

The online version contains supplementary material available at 10.1007/s00432-026-06524-7.

## Introduction

Lung cancer remains the leading cause of cancer-related mortality in Germany, with incidence among women continuing to rise, largely reflecting historical and ongoing patterns of tobacco consumption (Frost et al. 2022). Beyond active smoking, residential radon exposure is recognised as one of the leading environmental risk factors for lung cancer, affecting both smokers and never-smokers (Gridelli et al. 2015; Schabath and Cote 2019), with a well-established synergistic interaction with tobacco smoke (Barros-Dios et al. 2002, 2012; Darby et al. 2006). Radon is a radioactive noble gas originating from the decay of uranium in soil and rock. It has been recognised as a human lung carcinogen since the late 1980s, following evaluations by the International Agency for Research on Cancer (IARC) and the U.S. Environmental Protection Agency (EPA) (IARC 1988; USEPA 1987). Upon inhalation, radon progeny such as polonium-218 and polonium-214 deposit in the bronchial epithelium. There the emission of high-energy α-particles induces complex DNA damage, genomic instability and altered gene expression, thereby contributing to lung carcinogenesis (Jia et al. 2021; Enjo-Barreiro et al. 2022).

Strong evidence for the carcinogenicity of radon originates from occupational studies of uranium miners (Loomis et al. 2018; Richardson et al. 2022). For German uranium miners who commenced employment after 1960 and were therefore exposed to lower radon concentrations relative to earlier cohorts, a statistically significant linear association between cumulative radon exposure and lung cancer mortality has been demonstrated, with effect modification by attained age and time since exposure (Kreuzer et al. 2015, 2023). These miners were employed by Wismut, a state-owned uranium mining company operating in the former German Democratic Republic, primarily in the Ore Mountains region (Erzgebirge). High underground radon concentrations occurred predominantly in the early years of uranium mining, prior to the implementation of radiation protection measures. In the German cohort of uranium miners first employed in 1960 or later and the international pooled analysis of uranium miners, several hundred lung cancer deaths were observed and analysed in relation to occupational radon exposure, underscoring the relevance of radon even at lower exposure levels (Kreuzer et al. 2015; Richardson et al. 2022). Complementing the occupational evidence, more recent pooled and case-control analyses continue to support the association between residential radon exposure and lung cancer risk (Lorenzo-Gonzalez et al. 2020).

In Germany, Thuringia and Saxony are characterised by elevated geogenic radon potential due to natural uranium deposits and legacy mining activities. Large parts of these regions have therefore been designated as radon precautionary areas, where preventive measures are legally mandated under German radiation protection regulations (Kümmel et al. 2014; Dushe et al. 2003; BfS 2026). In the general population of Germany, approximately 2800 lung cancer deaths per year—around 6% of all lung cancer deaths—are attributable to residential radon exposure. In the federal states of Thuringia and Saxony, the percentage of lung cancer cases due to radon exposure has been estimated at approximately 10.0% and 9.5%, respectively, reflecting the higher geogenic radon potential in these regions (Heinzl et al. 2024).

Lung cancer comprises a heterogeneous group of diseases with distinct biological and clinical characteristics. Small cell lung cancer (SCLC) is characterised by aggressive behaviour and early dissemination. Most cases, however, are classified as non-small cell lung cancer (NSCLC), among which adenocarcinoma has become the predominant subtype particularly among women and never-smokers (RKI 2026; Sher et al. 2008; Couraud et al. 2012). Environmental exposures, including radon and ambient fine particulate matter, have been established as causes of lung cancer by etiological studies and IARC analyses, and may also contribute to shifting histological patterns observed in recent decades (IARC 2012, 2016).

This is the context of the present retrospective, registry-based ecological study, which uses the federal state of Thuringia as a test case for exploring whether population-level differences in both estimated annual lung cancer incidence rates and tumour characteristics can be identified between communities with differing radon exposure. Given the ecological design and the absence of individual smoking and occupational exposure data, the study is intended to be descriptive and hypothesis-generating. In addition, we seek to determine whether the observed patterns are helpful in informing considerations for primary radon mitigation and future risk-adapted secondary prevention strategies.

## Materials and methods

### Radon exposure in Thuringian communities

**Fig. 1:**
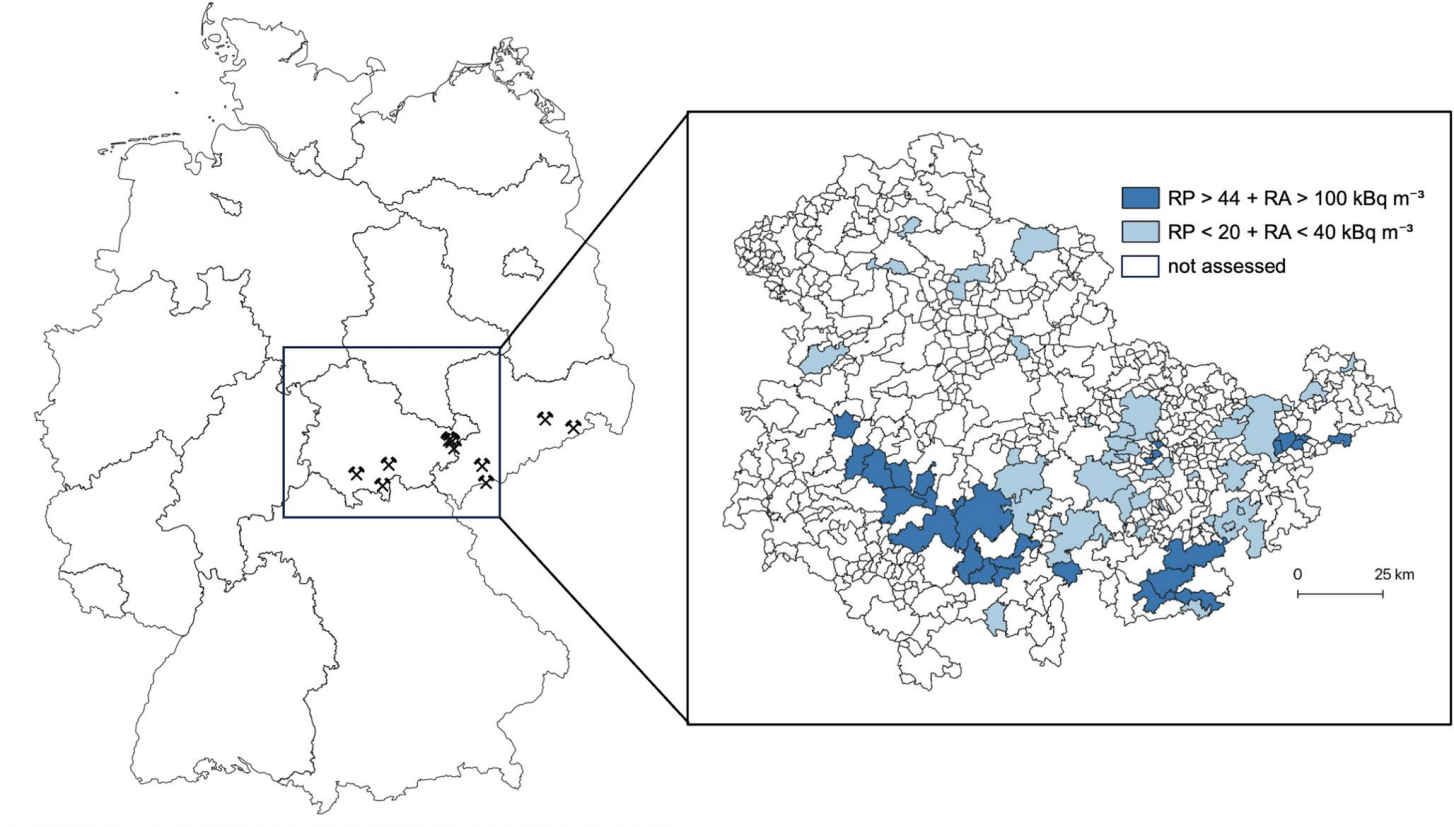
Spatial distribution of radon exposure levels in Thuringia (Germany)Spatial distribution of radon exposure levels in Thuringia (Germany). Left: Overview map of Germany with a frame marking the study region (Thuringia). Crossed-hammer symbols indicate former uranium mining sites operated by Wismut AG in Thuringia and Saxony. Right: Community-level classification in Thuringia based on radon potential (RP) and radon activity (RA) in soil gas. Communities were classified as high-radon exposure areas when both RP > 44 and RA > 100 kBq m⁻³ (dark blue), and as low-radon exposure areas when both RP < 20 and RA < 40 kBq m⁻³ (light blue). Regions with inconsistent RP or RA values were not assessed because they did not meet the predefined inclusion criteria (white).

Radon exposure was defined using two parameters: geogenic radon potential (RP) and radon activity (RA). The methodology used to map the geogenic RP in Germany has been described previously (Kemski et al. 2001). Geogenic RP was assessed by the Thuringian State Office for the Environment, Mining and Nature Conservation in most communities in the federal state of Thuringia (TLUBN 2025c). The radon activity concentration in soil air was measured in Thuringian communities as part of a monitoring program conducted by both the Federal Office for Radiation Protection (BfS) and by the Thuringian State Office for the Environment, Mining and Nature Conservation (TLUBN 2025b). As geogenic radon potential is primarily determined by stable geological conditions, temporal variability between measurements by BfS and TLUBN is limited. For this study, any community with an RP > 44 and an RA > 100 kBq m⁻³ was classified as having high-radon exposure (Supplemental Table 1). In contrast, communities with an RP < 20 and measured RA < 40 kBq m⁻³ were classified as having low-radon exposure (Supplemental Table 2). Communities with intermediate RP or RA values that did not meet the predefined thresholds for either high or low exposure were excluded from the comparative analysis to maximize contrast between exposure groups. Spatial patterns of radon exposure across the study region were visualised using GIS-based mapping (Geographic Information System), integrating community-level boundaries with geogenic RP and soil RA data (Fig. 1).

### Patients and tumour characteristics

**Fig. 2:**
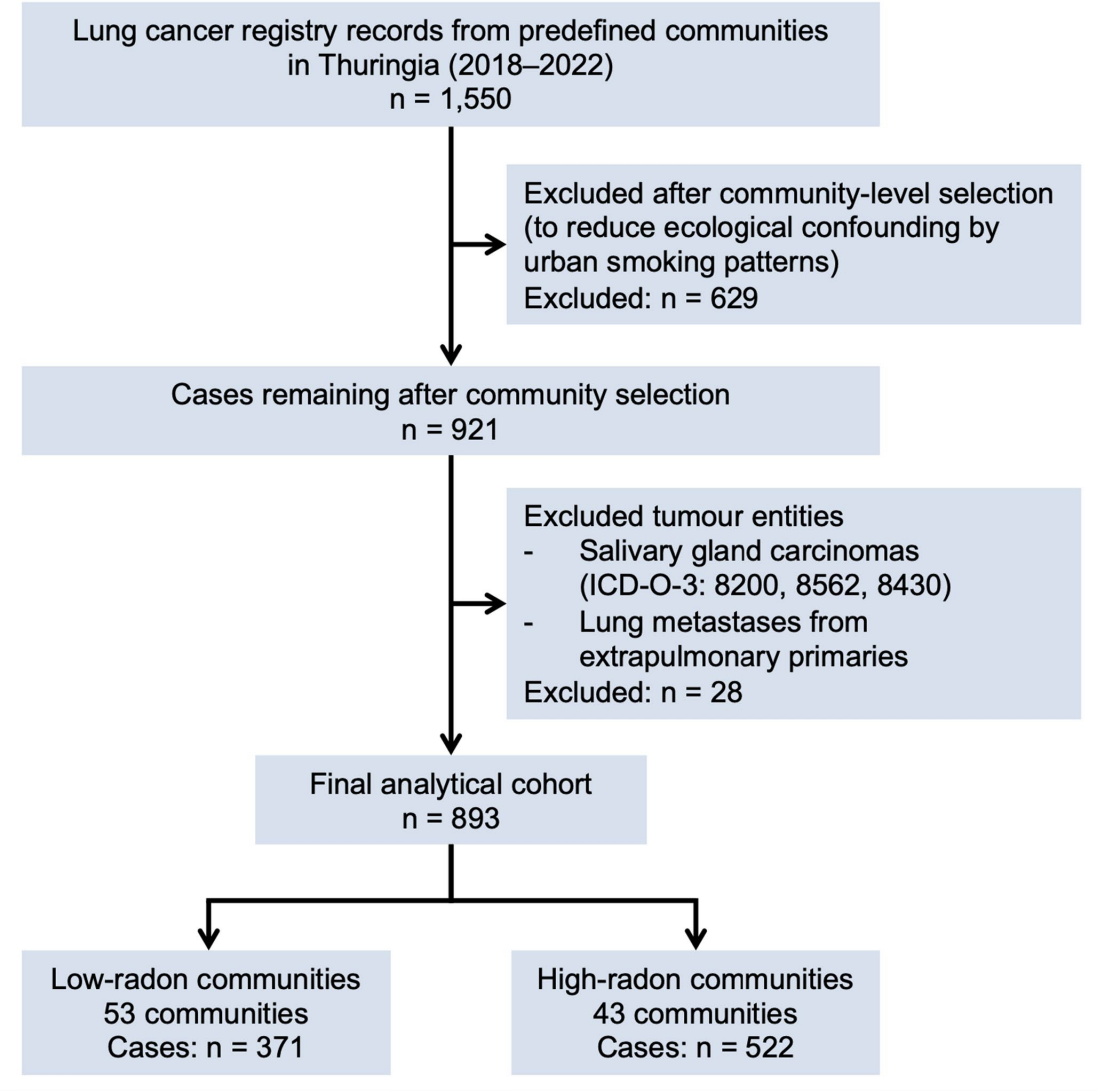
Flow diagram of case selection for the ecological analysis. From 1550 registry cases identified in predefined Thuringian communities between 2018 and 2022, cases from high-density urban municipalities were excluded (*n* = 629). Among the remaining 921 cases, salivary gland carcinomas and lung metastases from extrapulmonary primary tumours were excluded (*n* = 28), resulting in a final analytical study population of 893 patients from 96 communities (53 low-radon and 43 high-radon communities).

The State Cancer Registry of Thuringia provided anonymised data on patients from predefined communities with either high- or low-radon exposure diagnosed with lung cancer between 2018 and 2022. The data set contained information on 1,550 cases. After excluding communities with a high population density – in order to minimise ecological fallacy due to the higher smoking rates in large cities (Lubin 2002; Völzke et al. 2006) – 921 cases remained for the first analysis. Patients with lung carcinoma, classified according to the International Classification of Diseases for Oncology (ICD-O) with the localisation code C34, were included in the analysis. Further selection was based on ICD-O-3 morphology codes as defined by the US Centers for Disease Control and Prevention, including sarcomatoid tumours (8022, 8031–8033, 8972, 8980). After excluding salivary gland carcinomas (8200, 8562, 8430) and metastases of the lung with extrapulmonary primary tumours, 893 cases remained for the final analysis **(**Fig. 2**)**. Tumour stages were defined using the 8th edition of the Union for International Cancer Control (UICC) TNM. Population data for the communities analysed in this study were obtained from the database of the Thuringian State Office for Statistics (TLS 2026). Data processing was performed in accordance with the Declaration of Helsinki.

### Statistical analysis

Age was compared between radon exposure groups using the Mann–Whitney U test. Associations between categorical variables and radon exposure were explored using chi-square (χ²) tests based on contingency tables. For variables with multiple categories (e.g., histological subtypes or disease stages), overall distributional comparisons were considered primary, whereas row-wise subgroup comparisons were treated as descriptive. To account for multiple comparisons in these subgroup analyses, p-values were adjusted using the Benjamini–Hochberg false discovery rate procedure; adjusted p-values < 0.01 were considered statistically significant, whereas unadjusted p-values between 0.01 and 0.05 were regarded as nominal. Effect sizes were quantified using Cramer’s V and interpreted descriptively.

Annual incidence rates were estimated from cases (2018–2022) divided by five, using the 2022 population as denominator. Population-level incidence was compared between high- and low-radon communities using Poisson regression with aggregated case counts as the dependent variable and the log population (×5 years) as offset. Models included radon exposure (unadjusted) and additionally age group (< 60, 61–74, > 74 years) and sex (adjusted). Results are reported as incidence rate ratios (IRR) with 95% confidence intervals. All tests were two-sided. Analyses were performed using GraphPad Prism (version 8.2.0) and SPSS (version 31.0.1.0).

## Results

### Study population characteristics, age and sex distribution

**Fig. 3:**
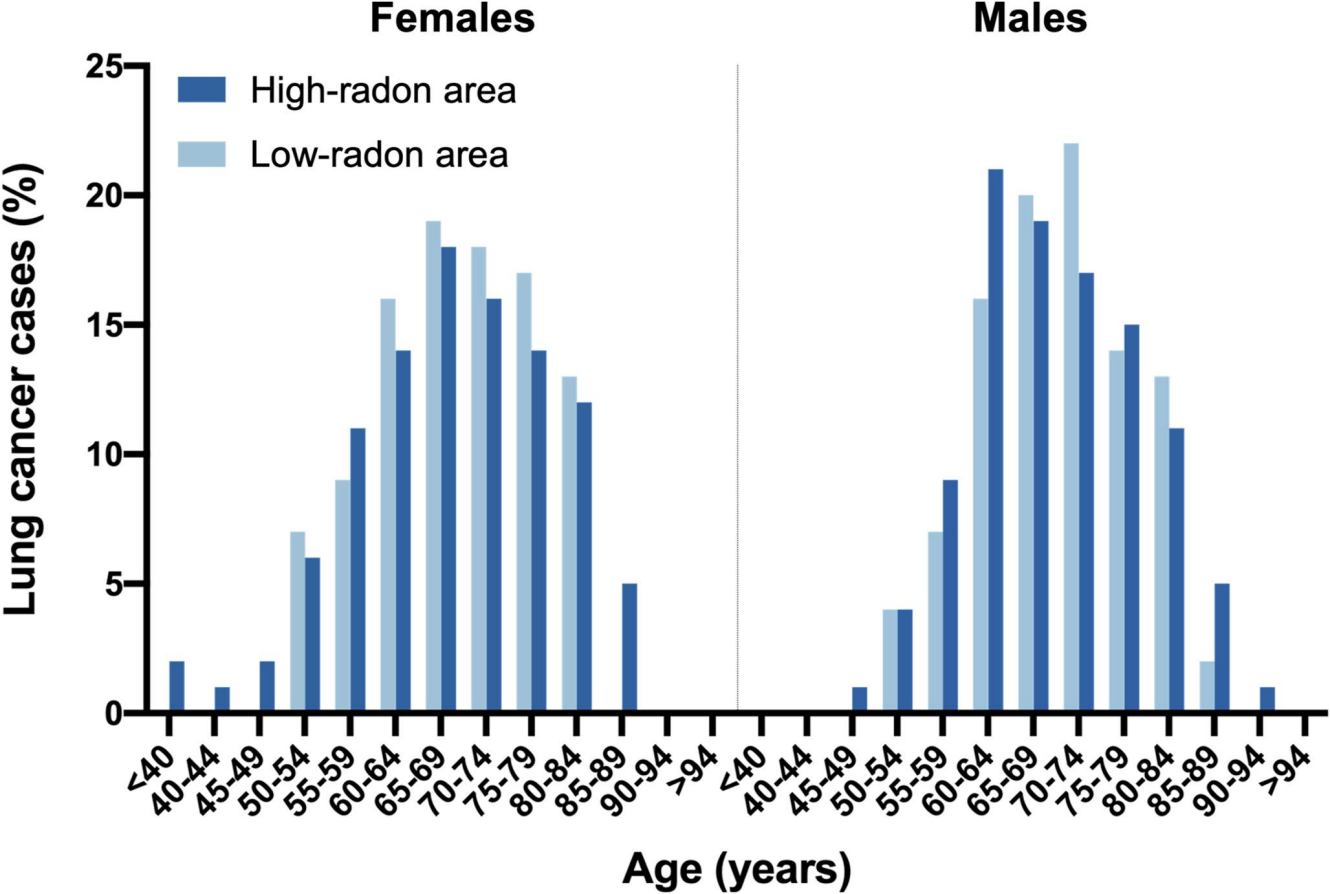
Age distribution of lung cancer cases among females and males in Thuringia according to residential radon exposure. Shown are relative frequencies of lung cancer cases in different age groups for individuals with high-radon exposure (dark blue) compared to those with low-radon exposure (light blue). Data source: Thuringian Cancer Registry (2018–2022).

Based on survey data from the Thuringian State Office for the Environment, Mining and Nature Conservation, 43 communities in Thuringia with high-radon and 53 communities with low-radon exposure were identified **(**Fig. 1, Supplemental Tables 1 and 2). Data from a total of 893 patients with lung cancer (*n* = 371 in low radon exposure areas, *n* = 522 in high-radon exposure areas) reported to the State Cancer Registry of Thuringia from 2018 to 2022 were analysed. Median age was identical in both groups (69 years) and did not differ significantly between radon exposure groups (Mann–Whitney U test, *p* = 0.796). The sex distribution did not differ significantly between groups, with 68% of patients in low-exposure and 67% in high-exposure areas being male (χ^2^ = 0.32, *p* = 0.57, Cramer’s V = 0.02) **(**Table 1**)**. In the female study population, the median age at diagnosis was similar in both exposure groups; however, the age distribution was broader in regions with higher radon exposure. Notably, 4.6% of cases occurred in women younger than 50 years in high-radon areas, whereas no cases were observed in these age groups in low-radon regions (Fig. 3).

**Table 1:** Demographic and clinical characteristics of lung cancer according to radon exposure in the area of residence in Thuringia (*n* = 893)

Characteristics	Total	Low-radon area	High-radon area	*p*-value
	*n* = 893	*n* = 371	*n* = 522	
Median age, years (range)	69 (34–94)	69 (44–94)	69 (34–91)	0.79
Sex, female (%)	291 (33)	117 (32)	174 (33)	0.57
*Subgroup, n (%)*				
NSCLC	667 (75)	278 (75)	389 (75)	0.89
Female	195 (29)	83 (30)	112 (29)	0.74
SCLC	190 (21)	80 (22)	110 (21)	0.46
Female	69 (36)	27 (33)	42 (38)	0.67
Other	36 (4)	13 (3)	23 (4)	0.50
*Grading, n (%)*				
G1	32 (4)	12 (3)	20 (4)	0.63
G2	197 (22)	76 (20)	121 (23)	0.34
G3	346 (39)	139 (37)	207 (40)	0.51
G4	46 (5)	21 (6)	25 (5)	0.56
n.a.	272 (30)	123 (33)	149 (29)	0.14
*NSCLC UICC stage, n (%)*				
IA1	4 (1)	2 (1)	2 (1)	0.73
IA2	10 (1)	6 (2)	4 (1)	0.24
IA3	15 (2)	4 (1)	11 (3)	0.23
IB	11 (2)	5 (2)	6 (2)	0.79
IIA	15 (2)	6 (2)	9 (2)	0.88
IIB	21 (3)	13 (5)	8 (2)	0.06
IIIA	57 (9)	26 (9)	31 (8)	0.53
IIIB	66 (10)	24 (9)	42 (11)	0.35
IIIC	41 (6)	20 (7)	21 (5)	0.34
IVA	115 (17)	48 (17)	67 (17)	0.94
IVB	202 (30)	84 (30)	118 (30)	0.94
n.a.	110 (16)	40 (14)	70 (18)	0.21
*SCLC UICC stage, n (%)*				
Very limited disease	5 (3)	1 (1)	4 (4)	0.30
Limited disease	21 (11)	13 (16)	8 (7)	0.05
Extensive disease	109 (57)	39 (49)	70 (64)	0.04
n.a.	55 (29)	27 (34)	28 (25)	0.21

NSCLC, non-small cell lung cancer; SCLC, small cell lung cancer; UICC, Union for International Cancer Control; G, histological grade; n.a., not available

### Overall lung cancer histology and stage

With respect to histological subtypes, NSCLC predominated and accounted for 75% of all cases, whereas SCLC represented 21%. The proportion of NSCLC (75% in both groups, *p* = 0.89) and SCLC (22% vs. 21%, *p* = 0.46) did not differ significantly between exposure groups, nor were there differences in histological grading or NSCLC UICC stage. However, among SCLC patients, stage distribution varied by radon exposure: nominal differences in SCLC stage distribution were observed in unadjusted analyses in patients from high-radon areas compared with low-radon areas (64% vs. 49%, χ^2^ = 4.24, *p* = 0.04, Cramer’s V = 0.07), but these associations did not remain significant after correction for multiple testing. Limited disease was observed nominally more often in low-radon regions (16% vs. 7%, χ^2^ = 3.84, *p* = 0.05, Cramer’s V = 0.06) **(**Table 1**)**. Missing data proportions for grading and stage variables did not differ significantly between low- and high-radon groups (grading: 33% vs. 29%, *p* = 0.14; NSCLC stage: 14% vs. 18%, *p* = 0.21; SCLC stage: 34% vs. 25%, *p* = 0.21), suggesting no clear evidence of differential missingness by exposure group.

### Lung cancer subtype variation

Among the 667 patients with NSCLC, adenocarcinoma was the most common subtype overall (53%), followed by squamous cell carcinoma (39%). When stratified by radon exposure, adenocarcinoma was more frequent in patients from high-radon areas compared with low-radon areas (56% vs. 48%, χ^2^ = 4.28, *p* = 0.04, Cramer’s V = 0.07). Several squamous cell carcinoma subtypes were observed more frequently in low-radon regions. A nominal difference in keratinising squamous cell carcinoma distribution was observed (6% vs. 3%; χ² = 4.39, *p* = 0.04, Cramer’s V = 0.07), but this finding was not retained after false discovery rate correction. Among histological subgroup analyses, only non-keratinising large cell squamous cell carcinoma remained statistically significant after false discovery rate correction (10% vs. 3%; χ² = 11.69, *p* < 0.001, Cramer’s V = 0.11) (Table 2). Of 190 patients with SCLC, the majority were classified as SCLC without further specification (83%), with similar proportions in low- and high-radon areas (89% vs. 78%, χ^2^ = 3.64, *p* = 0.06, Cramer’s V = 0.06). A notable difference was observed for combined small cell carcinoma, which occurred exclusively in patients from high-radon regions (7% vs. 0%, χ^2^ = 6.12, *p* = 0.01, Cramer’s V = 0.08). Other subtypes, including carcinoid tumours (13% overall), atypical carcinoid tumours (5%) and neuroendocrine carcinomas (2%), showed no significant variation by radon exposure (Table 3).

**Table 2:** NSCLC subtypes according to radon exposure in the area of residence in Thuringia

ICD-O-3 histological subtypes	Total	Low-radon area	High-radon area	*p*-value
	*n* = 667	*n* = 278	*n* = 389	
Squamous cell carcinoma (SCC), n (%)	262 (39)	115 (41)	147 (38)	0.35
Malignant epithelial carcinoma, n (%)	35 (5)	13 (5)	22 (6)	0.58
Epidermoid carcinoma, n (%)	160 (24)	59 (21)	101 (26)	0.16
Large cell SCC, keratinising, n (%)	26 (4)	16 (6)	10 (3)	0.04
Large cell SCC, non-keratinising, n (%)	40 (6)	27 (10)	13 (3)	< 0.01
Basaloid SCC, n (%)	1 (0)	0 (0)	1 (0)	0.40
Adenocarcinoma (AC), n (%)	353 (53)	134 (48)	219 (56)	0.04
AC, without further specification, n (%)	315 (47)	122 (44)	193 (50)	0.14
Solid AC with mucus formation	9 (1)	4 (1)	5 (1)	0.87
Non-mucinous bronchiolo-alveolar AC	10 (0)	3 (1)	7 (2)	0.45
Mucinous bronchiolo-alveolar AC	8 (1)	3 (1)	5 (1)	0.81
AC with mixed subtypes	1 (0)	1 (0)	0 (0)	0.24
Acinar AC	1 (0)	0 (0)	1 (0)	0.40
Large cell lung carcinoma (LCLC), n (%)	23 (3)	14 (5)	9 (2)	0.06
LCLC, without further specification, n (%)	6 (1)	5 (2)	1 (0)	0.04
Large cell neuroendocrine carcinoma, n (%)	17 (3)	9 (3)	8 (2)	0.34
Adenosquamous carcinoma, n (%)	12 (2)	4 (1)	8 (2)	0.55
Other, n (%)	17 (3)	11 (4)	6 (2)	0.05
Undifferentiated carcinoma, n (%)	2 (0)	2 (1)	0 (0)	0.09
Non-small cell carcinoma, n (%)	15 (2)	9 (3)	6 (2)	0.15

ICD-O-3, International Classification of Diseases for Oncology, Third Edition; SCC, squamous cell carcinoma; AC, adenocarcinoma; LCLC, large cell lung carcinoma.

**Table 3:** SCLC subtypes according to radon exposure in the area of residence in Thuringia

ICD-O-3 histological subtypes	Total	Low-radon area	High-radon area	*p*-value
	*n* = 190	*n* = 80	*n* = 110	
SCLC, without further specification, n (%)	157 (83)	71 (89)	86 (78)	0.06
Combined small cell carcinoma, n (%)	8 (4)	0 (0)	8 (7)	0.01
Small cell carcinoma of the intermediate type, n (%)	1 (1)	0 (0)	1 (1)	0.39
Carcinoid tumour, without further specification, n (%)	24 (13)	9 (11)	15 (14)	0.62
Atypical carcinoid tumour, n (%)	10 (5)	6 (8)	4 (4)	0.24
Neuroendocrine tumour of the lung, n (%)	1 (1)	0 (0)	1 (1)	0.39
Neuroendocrine carcinoma of the lung, n (%)	4 (2)	0 (0)	4 (4)	0.08

ICD-O-3, International Classification of Diseases for Oncology, Third Edition; SCLC, small cell lung cancer

### Population-level lung cancer incidence rates

Across the study region, the estimated annual lung cancer incidence was 50.8 cases per 100,000 person-years, with similar rates in low-radon areas (49.5 per 100,000) and high-radon areas (51.8 per 100,000). In an unadjusted Poisson regression model using population size as offset term, residential radon exposure was not associated with lung cancer incidence (IRR 1.04, 95% CI 0.95–1.14, *p* = 0.414). In an additional model adjusted for age group and sex, results remained materially unchanged, with no significant association between high residential radon exposure and lung cancer incidence (adjusted IRR 1.05, 95% CI 0.92–1.20, *p* = 0.473). Age group and sex were significant independent predictors of incidence (both *p* < 0.001) (Table 4).

**Table 4:** Lung cancer incidence and Poisson regression analysis according to residential radon exposure in Thuringia (2018–2022)

Population (2022)	Total	Low-radon area	High-radon area
	351,451	149,795	201,656
Lung cancer cases	893	371	522
Incidence per 100,000 person-years	50.8	49.5	51.8

Poisson regression models			
Predictor	IRR	95% CI	*p*-value
High vs. low radon (unadjusted)	1.04	0.95–1.14	0.414
High vs. low radon (adjusted for age, sex)	1.05	0.92–1.20	0.473

NSCLC, non-small cell lung cancer; SCLC, small cell lung cancer; IRR, incidence rate ratio

## Discussion

This population-based, registry-driven ecological analysis of lung cancer in Thuringia provides exploratory insights into potential associations between geogenic radon exposure and tumour characteristics. While the estimated annual incidence rates did not differ significantly between communities with low- and high-radon exposure, histological patterns shifted toward a higher proportion of adenocarcinoma in areas with high radon potential. Moreover, SCLC patients more often presented with extensive-stage disease, and women showed a broader age distribution, with cases under 50 years occurring exclusively in high-radon communities. The relatively high proportion of SCLC (21%) may reflect study population selection and regional smoking patterns. Given the ecological design, these findings should be interpreted as hypothesis-generating rather than causal.

The absence of differences in the estimated annual incidence rates between communities with contrasting radon levels appears counterintuitive, given the well-established dose–response relationship between residential radon exposure and lung cancer risk, with an estimated 16% increase in relative risk per 100 Bq m⁻³ increase in radon concentration (Darby et al. 2006). However, the ecological exposure definition applied in this study—based on community-level radon potential and soil radon activity—is inherently prone to non-differential exposure misclassification, which tends to attenuate contrasts in the estimated annual lung cancer incidence rates at the population level. In addition, radon increases baseline lung cancer risk, particularly among smokers, with the joint effect of radon and tobacco exposure generally considered submultiplicative, and regional heterogeneity in tobacco use, socioeconomic factors and healthcare access is likely to outweigh radon-specific effects when aggregated to community-level estimates of annual lung cancer incidence (Tomasek 2013; IARC 2012). Recent analyses of uranium miner cohorts, including the German cohort of miners first employed in 1960 or later, have demonstrated linear radon–lung cancer associations as well as effect modification by attained age and time since exposure even at lower exposure levels, underscoring the complexity of translating individual-level risks to ecological settings (Loomis et al. 2018; Kreuzer et al. 2023).

Histological stratification revealed a higher proportion of adenocarcinoma among patients residing in areas with high-radon exposure. This observation is consistent with reports suggesting that adenocarcinoma and SCLC may be relatively sensitive to radon exposure. Importantly, a case-control study based on individual residential radon measurements reported differences in lung cancer characteristics among never-smokers and supported a possible association between radon exposure and adenocarcinoma-related patterns (Torres-Duran et al. 2015). However, systematic reviews and meta-analyses have yielded inconclusive results regarding histology-specific associations (Li et al. 2020; Ramkissoon et al. 2018). Globally, adenocarcinoma incidence has increased, particularly among never-smokers, with accumulating evidence that environmental factors such as radon and ambient fine particulate matter (PM₂․₅) may act as co-drivers. Mechanistic studies have demonstrated that PM₂․₅ can promote EGFR- and KRAS-driven adenocarcinogenesis via inflammation-mediated expansion of pre-existing oncogenic clones (Hill et al. 2023; Berg et al. 2023). Given that former mining regions may be characterised by elevated radon concentrations and that combined environmental exposures can vary regionally, the histological patterns observed in this ecological analysis may reflect multiple environmental influences rather than radon-specific effects alone.

The finding that extensive-stage disease was more frequent among SCLC patients from communities with high-radon exposure warrants careful interpretation. Epidemiological studies have reported an association between radon exposure and SCLC risk (Field et al. 2000; Ramkissoon et al. 2018). Importantly, the only case-control study conducted exclusively in patients with SCLC also supported an association between residential radon exposure and SCLC risk (Rodriguez-Martinez et al. 2022). However, smoking is both the dominant risk factor for SCLC and a well-established synergistic effect modifier of radon-associated lung cancer risk, with evidence for submultiplicative interaction (Richardson et al. 2022). Experimental data indicate that high-linear-energy-transfer α-particle radiation induces complex DNA damage and genomic instability, biological mechanisms that may contribute to aggressive tumour behaviour (BEIR-IV. 1988; Jia et al. 2021). Genome-wide analyses of uranium miners have linked radon-associated lung cancer to alterations in genes such as *CHRNB4*, a neuroendocrine driver implicated in SCLC aggressiveness (Rosenberger et al. 2018; Peinado et al. 2025). Nevertheless, the absence of smoking data represents a major limitation and precludes any inference regarding the independent contribution of radon exposure. Notably, lung cancer cases in women younger than 50 years of age were observed exclusively in communities classified as having high-radon exposure. This pattern parallels the global epidemiological trend of rising lung adenocarcinoma incidence among younger, never-smoking women (LoPiccolo et al. 2024).

Epidemiological studies integrating molecular markers have reported a predominance of specific alterations, including *EGFR* mutations and *ALK* rearrangements, in this subgroup (Ruano-Ravina et al. 2016). Whether environmental exposures such as radon contribute to the enrichment of these molecular phenotypes remains speculative and warrants investigation in studies integrating molecular tumour profiling with individual exposure assessment.

This study benefits from comprehensive cancer registry data, standardised histological classification and TNM-based staging, as well as an exposure classification aligned with German regulatory frameworks for radon precautionary areas (BfS 2025; TLUBN 2025a). However, several limitations must be emphasised. First, this was an ecological analysis based on community-level exposure indicators rather than individual residential radon measurements, making exposure misclassification likely. In particular, no information was available on the specific dwelling characteristics of lung cancer cases, such as floor level, building type, basement contact, ventilation, renovation status or residential mobility. Consequently, individuals living on upper floors in communities classified as high-radon areas may have experienced substantially lower indoor radon exposure than assumed. Such non-differential misclassification could likely attenuate true associations. Additional limitations include the absence of smoking and occupational exposure data, potential selection bias introduced by the exclusion of large urban municipalities and limited statistical power in rare histological subgroups. Recently published population-based radon exposure maps for Germany provide spatially resolved estimates that are more directly applicable to epidemiological research and may enable refined exposure modelling and sensitivity analyses in future studies (Petermann and Hoffmann 2025). Accordingly, causal inference is not possible, and findings should be interpreted with appropriate caution.

From a public health perspective, these exploratory findings reinforce the importance of radon measurement and mitigation in designated precautionary areas where geogenic radon potential exceeds regulatory thresholds. Structural measures such as basement sealing, pressure-controlled ventilation and long-term detector programs remain cornerstones of primary prevention. The observed enrichment of adenocarcinoma and broader age distribution in high-radon communities may also support targeted awareness initiatives, particularly among women and never-smokers.

In conclusion, this population-based ecological study suggests that while residential radon exposure may not substantially influence the estimated annual lung cancer incidence rates at the community level, it is associated with differences in tumour histology, stage at diagnosis and age distribution in women. Given the ecological design, potential exposure misclassification and lack of individual smoking data, these findings should be regarded as hypothesis-generating. In addition, the observed effect sizes quantified by Cramer’s V were generally small (V ≈ 0.06–0.11), indicating weak associations despite statistical significance. This pattern is expected in large observational datasets, where modest distributional differences may reach statistical significance without implying strong biological effects. These findings highlight the need for future studies integrating individual radon measurements, detailed smoking histories and molecular tumour characterisation to clarify the role of radon—alone and in interaction with tobacco exposure—in lung cancer biology and prevention.

## Supplementary Information

Below is the link to the electronic supplementary material.

Supplementary Material 1

## Data Availability

For original data, please contact philipp.ernst@med.uni-jena.de.
