# Supplementary material for: Residential radon exposure and lung cancer histology and stage: a population-based ecological study in Central Germany
Source: J Cancer Res Clin Oncol. 2026 May 31;152(6):115. doi: 10.1007/s00432-026-06524-7 (PMC13230297; doi:10.1007/s00432-026-06524-7)
Supplement: Supplementary file 1 — Supplementary Material 1 [file 432_2026_6524_MOESM1_ESM.pdf]

Supplemental Material

Residential Radon Exposure and Lung Cancer Histology and Stage: A Population-Based Ecological Study in Central Germany

Philipp Ernst[1], Tobias Rachow[2], Sebastian Henn[3], Jakob Friedrich Hammersen[1], Silvio Dittrich[4], Annika Heßmer[5], Alexander Hillig[5], Astrid Heßmer[5] and Andreas Hochhaus[1]

[1] Klinik für Innere Medizin II, Universitätsklinikum Jena, Comprehensive Cancer Center Central Germany, Campus Jena, Jena, Germany

[2] IOGP MVZ GmbH, Gera, Germany

[3] Institut für Wirtschaftsgeographie, Friedrich-Schiller-Universität Jena, Jena, Germany

[4] Wismut-Erbe-Forschung, Sächsische Akademie für Wissenschaft, Leipzig, Germany

[5] Landeskrebsregister Thüringen, Jena, Germany

Key Words:

Lung cancer, epidemiology, radon exposure

Corresponding author:

Dr. med. Philipp Ernst, M.Sc.

Klinik für Innere Medizin II, Universitätsklinikum Jena

Am Klinikum 1, 07740 Jena, Germany

Tel: +49 3641-9324277

Fax: +49 3641 9324202

Email: philipp.ernst@med.uni-jena.de

Supplemental Table 1. Thuringian communities classified as high-radon exposure areas (n = 43).

Community	Measurement site	Year of measurement	Geological classification	Radon potential	Radon concentration (kBq/m^3)
Altenfeld	StSch 4106 Thüringer Wald 70	1996	Metasediment (metamorphosed siliciclastic rock)	81	>100
Bad Colberg-Heldburg	StSch 4106 Deutschland Ergänzung 252	2005	Keuper (Upper Triassic Marl/Claystone)	121	>100
Benshausen	StSch 4106 Thüringer Wald 33	1996	Basalt (Volcanic rock)	134	>100
Buchholz	StSch 4187 Harz/Südrand 24	1999	Zechstein (Upper Permian Evaporites)	64	>100
Crispendorf	StSch 4106 Vogtland 57	1996	Carboniferous	100	>100
Ebersdorf	StSch 4106 Vogtland 46	1996	Carboniferous	51	>100
Elgersburg	TH-76	2022	Rotliegend (Lower Permian Red Beds)	111	>100
Emsetal	StSch 4106 Thüringer Wald 19	1996	Rotliegend (Lower Permian Red Beds)	101	>100
Finsterbergen	StSch 4106 Thüringer Wald 24	1996	Volcanic rock (likely rhyolite or andesite)	210	>100
Floh-Seligenthal	StSch 4106 Thüringer Wald 30	1996	Rotliegend (Lower Permian Red Beds)	106	>100
Gefell	StSch 4106 Vogtland 50	1996	Ordovician	94	>100
Georgenthal	StSch 4106 Thüringer Wald 27	1996	Volcanic rock (likely rhyolite or andesite)	167	>100
Goldisthal	TH-68	2022	Volcanic rock (likely rhyolite or andesite)	53	>100
Gräfenthal	TH-58	2022	Siliciclastic sedimentary rocks	231	>100
Großbockedra	StSch 4062 Thüringer Becken/Süd 25	1993	Buntsandstein (Lower Triassic Sandstone)	60	>100
Großbreitenbach	TH-202	1999	Metasediment (metamorphosed siliciclastic rock)	76	>100
Günserode	StSch 4187 Harz/Südrand 36	1999	Keuper (Upper Triassic Marl/Claystone)	68	>100
Heinersdorf	StSch 4106 Vogtland 35	1996	Carboniferous	92	>100
Ilmenau	StSch 4106 Thüringer Wald 64	1996	Basalt (Volcanic rock)	107	>100
Katzhütte	StSch 4106 Thüringer Wald 80	1996	Metasediment (metamorphosed siliciclastic rock)	87	>100
Kauern	TH-16	2022	Siliciclastic sedimentary rocks	115	>100
Kleinschmalkalden	StSch 4106 Thüringer Wald 23	1996	Rotliegend (Lower Permian Red Beds)	101	>100
Lobenstein	StSch 4106 Vogtland 47	1996	Ordovician	105	>100
Luisenthal	StSch 4106 Thüringer Wald 47	1996	Volcanic rock (likely rhyolite or andesite)	90	>100
Mannstedt	StSch 4062 Thüringer Becken/Süd 60	1995	Keuper (Upper Triassic Marl/Claystone)	51	>100
Marksuhl	StSch 4106 Thüringer Wald 4	1996	Rotliegend (Lower Permian Red Beds)	96	>100
Masserberg	TH-84	2022	Metasediment (metamorphosed siliciclastic rock)	92	>100
Moorgrund	StSch 4106 Thüringer Wald 9	1996	Rotliegend (Lower Permian Red Beds)	65	>100
Oberhof	StSch 4106 Thüringer Wald 44	1996	Volcanic rock (likely rhyolite or andesite)	144	>100
Paitzdorf	TH-42	2022	Siliciclastic sedimentary rocks	129	>100
Ponitz	StSch 4251 Erzgebirge/Süd 39	2003	Zechstein (Upper Permian Evaporites)	64	>100
Posterstein	TH-10	2022	Volcanic rock (likely rhyolite or andesite)	68	>100
Ronneburg	TH-07	2022	Siliciclastic sedimentary rocks	130	>100
Ruhla	StSch 4106 Thüringer Wald 11	1996	Metasediment (metamorphosed siliciclastic rock)	112	>100
Schleiz	StSch 4106 Vogtland 59	1996	Siliciclastic sedimentary rocks	93	>100
Schmiedefeld am Rennsteig	StSch 4106 Thüringer Wald 60	1996	Plutonic rock (likely granitic)	135	>100
Seitenroda	TH-56	2024	Siliciclastic sedimentary rocks	641	>100
Steinbach-Hallenberg	StSch 4106 Thüringer Wald 34	1996	Basalt (Volcanic rock)	197	>100
Stützerbach	StSch 4106 Thüringer Wald 62	1996	Volcanic rock (likely rhyolite or andesite)	99	>100
Suhl	StSch 4106 Thüringer Wald 55	1996	Volcanic rock (likely rhyolite or andesite)	147	>100
Tambach-Dietharz	StSch 4106 Thüringer Wald 42	1996	Rotliegend (Lower Permian Red Beds)	174	>100
Trusetal	StSch 4106 Thüringer Wald 18	1996	Plutonic rock (likely granitic)	117	>100
Unterbodnitz	StSch 4062 Thüringer Becken/Süd 24	1993	Buntsandstein (Lower Triassic Sandstone)	68	>100

StSch — Radon monitoring station operated by the Federal Office for Radiation Protection (BfS). The site is equipped with a borehole-installed probe using a packer-based measurement technique for isolated gas extraction and continuous radon concentration monitoring.

TH — Regional radon monitoring site operated by the Thuringian State Office for the Environment, Mining and Nature Conservation (TLUBN) according to DIN ISO 11665-11 (Measurement of radon activity concentration – Method for determination of soil gas concentration).

Supplemental Table 2. Thuringian communities classified as low-radon esposure areas (n = 53).

Community	Measurement site	Year of measurement	Geological classification	Radon potential	Radon concentration (kBq/m^3)
Altenberga	StSch 4062 Thüringer Becken/Süd 3	1993	Muschelkalk (Middle Triassic Limestone)	11	<40
Altkirchen	StSch 4251 Erzgebirge/Süd 32	2003	Quaternary	12	<40
Bad Frankenhausen	StSch 4187 Harz/Südrand 35	1999	Zechstein (Upper Permian Evaporites)	10	<40
Bad Köstritz	StSch 4251 Erzgebirge/Süd 40	2003	Buntsandstein (Lower Triassic Sandstone)	14	<40
Badra	StSch 4187 Harz/Südrand 29	1999	Zechstein (Upper Permian Evaporites)	14	<40
Birkigt	StSch 4251 Thüringer Becken/Nord 21	2001	Buntsandstein (Lower Triassic Sandstone)	8	<40
Bösleben-Wüllersleben	StSch 4251 Thüringer Becken/Nord 43	2001	Muschelkalk (Middle Triassic Limestone)	18	<40
Bucha	StSch 4062 Thüringer Becken/Süd 20	1993	Muschelkalk (Middle Triassic Limestone)	13	<40
Creuzburg	StSch 4251 Thüringer Becken/Nord 30	2001	Muschelkalk (Middle Triassic Limestone)	13	<40
Ebenheim	StSch 4251 Thüringer Becken/Nord 28	2001	Muschelkalk (Middle Triassic Limestone)	14	<40
Föritz	StSch 4106 Vogtland 135	1996	Quaternary	3	<40
Gera	StSch 4251 Erzgebirge/Süd 24	2003	Buntsandstein (Lower Triassic Sandstone)	14	<40
Gössitz	StSch 4106 Vogtland 66	1996	Carboniferous	16	<40
Großenehrich	StSch 4187 Harz/Südrand 28	1999	Muschelkalk (Middle Triassic Limestone)	6	<40
Großlohra	StSch 4187 Harz/Südrand 14	1999	Buntsandstein (Lower Triassic Sandstone)	9	<40
Großrudestedt	StSch 4251 Thüringer Becken/Nord 34	2001	Keuper (Upper Triassic Marl/Claystone)	8	<40
Gumperda	StSch 4062 Thüringer Becken/Süd 13	1993	Muschelkalk (Middle Triassic Limestone)	17	<40
Harra	StSch 4106 Vogtland 156	1996	Ordovician	6	<40
Herschdorf	StSch 4106 Thüringer Wald 73	1996	Metasediment (metamorphosed siliciclastic rock)	12	<40
Hirschberg	StSch 4106 Vogtland 106	1996	Basalt (Volcanic rock)	14	<40
Hohlstedt	StSch 4062 Thüringer Becken/Süd 39	1994	Muschelkalk (Middle Triassic Limestone)	19	<40
Hummelshain	StSch 4106 Vogtland 117	1996	Buntsandstein (Lower Triassic Sandstone)	5	<40
Jena	StSch 4062 Thüringer Becken/Süd 21	1993	Muschelkalk (Middle Triassic Limestone)	19	<40
Kamsdorf	StSch 4106 Vogtland 71	1996	Zechstein (Upper Permian Evaporites)	11	<40
Kaulsdorf	StSch 4106 Vogtland 16	1996	Zechstein (Upper Permian Evaporites)	9	<40
Kiliansroda	StSch 4062 Thüringer Becken/Süd 51	1994	Muschelkalk (Middle Triassic Limestone)	7	<40
Kirchhasel	StSch 4106 Vogtland 123	1996	Buntsandstein (Lower Triassic Sandstone)	18	<40
Königsee	StSch 4106 Vogtland 127	1996	Buntsandstein (Lower Triassic Sandstone)	13	<40
Kraftsdorf	StSch 4251 Thüringer Becken/Nord 26	2001	Buntsandstein (Lower Triassic Sandstone)	11	<40
Krölpa	StSch 4106 Vogtland 122	1996	Buntsandstein (Lower Triassic Sandstone)	10	<40
Linda	StSch 4106 Vogtland 8	1996	Carboniferous	9	<40
Menteroda	StSch 4251 Thüringer Becken/Nord 5	2001	Muschelkalk (Middle Triassic Limestone)	19	<40
Milda	StSch 4062 Thüringer Becken/Süd 1	1993	Muschelkalk (Middle Triassic Limestone)	4	<40
Pößneck	StSch 4106 Vogtland 121	1996	Buntsandstein (Lower Triassic Sandstone)	7	<40
Reinstädt	StSch 4062 Thüringer Becken/Süd 44	1994	Muschelkalk (Middle Triassic Limestone)	7	<40
Rosendorf	StSch 4106 Vogtland 114	1996	Buntsandstein (Lower Triassic Sandstone)	1	<40
Saalfeld	StSch 4106 Vogtland 128	1996	Buntsandstein (Lower Triassic Sandstone)	6	<40
Saalfelder Höhe	StSch 4106 Vogtland 109	1996	Ordovician	19	<40
Schalkau	StSch 4106 Vogtland 132	1996	Buntsandstein (Lower Triassic Sandstone)	16	<40
Schernberg	StSch 4187 Harz/Südrand 33	1999	Muschelkalk (Middle Triassic Limestone)	13	<40
Schleusegrund	StSch 4106 Thüringer Wald 84	1996	Basalt (Volcanic rock)	7	<40
Schmorda	StSch 4106 Vogtland 18	1996	Carboniferous	6	<40
Schömberg	StSch 4251 Erzgebirge/Süd 23	2003	Carboniferous	11	<40
Solkwitz	StSch 4106 Vogtland 12	1996	Zechstein (Upper Permian Evaporites)	16	<40
Stadtilm	StSch 4106 Vogtland 125	1996	Muschelkalk (Middle Triassic Limestone)	17	<40
Starkenberg	StSch 4251 Erzgebirge/Süd 34	2003	Quaternary	9	<40
Steinheid	StSch 4106 Vogtland 131	1996	Ordovician	14	<40
Sulza	StSch 4062 Thüringer Becken/Süd 26	1993	Buntsandstein (Lower Triassic Sandstone)	12	<40
Tegau	StSch 4106 Vogtland 20	1996	Carboniferous	12	<40
Treben	StSch 4251 Leipzig 3	2003	Quaternary	17	<40
Triebes	StSch 4251 Erzgebirge/Süd 19	2003	Ordovician	9	<40
Trockenborn-Wolfersdorf	StSch 4106 Vogtland 116	1996	Buntsandstein (Lower Triassic Sandstone)	5	<40
Vollenborn	StSch 4251 Thüringer Becken/Nord 2	2001	Buntsandstein (Lower Triassic Sandstone)	17	<40

StSch — Radon monitoring station operated by the Federal Office for Radiation Protection (BfS). The site is equipped with a borehole-installed probe using a packer-based measurement technique for isolated gas extraction and continuous radon concentration monitoring.
